# Detection of growth-related QTL in turbot (*Scophthalmus maximus*)

**DOI:** 10.1186/1471-2164-12-473

**Published:** 2011-09-29

**Authors:** Enrique Sánchez-Molano, Alex Cerna, Miguel A Toro, Carmen Bouza, Miguel Hermida, Belén G Pardo, Santiago Cabaleiro, Jesús Fernández, Paulino Martínez

**Affiliations:** 1 Departamento de Mejora Genética Animal, Instituto Nacional de Investigación y Tecnología Agraria y Alimentaria, Ctra. Coruña Km. 7.5. 28040 Madrid, Spain; 2 Departamento de Producción Animal, ETS Ingenieros Agrónomos, Universidad Politécnica de Madrid, Ciudad Universitaria, 28040 Madrid, Spain; 3 Departamento de Xenética, Universidade de Santiago de Compostela, Facultade de Veterinaria, 27002, Lugo, Spain; 4 Cluster de la Acuicultura de Galicia (CETGA), Punta de Couso s/n, 15965, Aguiño, Ribeira, Spain

## Abstract

**Background:**

The turbot (*Scophthalmus maximus*) is a highly appreciated European aquaculture species. Growth related traits constitute the main goal of the ongoing genetic breeding programs of this species. The recent construction of a consensus linkage map in this species has allowed the selection of a panel of 100 homogeneously distributed markers covering the 26 linkage groups (LG) suitable for QTL search. In this study we addressed the detection of QTL with effect on body weight, length and Fulton's condition factor.

**Results:**

Eight families from two genetic breeding programs comprising 814 individuals were used to search for growth related QTL using the panel of microsatellites available for QTL screening. Two different approaches, maximum likelihood and regression interval mapping, were used in order to search for QTL. Up to eleven significant QTL were detected with both methods in at least one family: four for weight on LGs 5, 14, 15 and 16; five for length on LGs 5, 6, 12, 14 and 15; and two for Fulton's condition factor on LGs 3 and 16. In these LGs an association analysis was performed to ascertain the microsatellite marker with the highest apparent effect on the trait, in order to test the possibility of using them for marker assisted selection.

**Conclusions:**

The use of regression interval mapping and maximum likelihood methods for QTL detection provided consistent results in many cases, although the high variation observed for traits mean among families made it difficult to evaluate QTL effects. Finer mapping of detected QTL, looking for tightly linked markers to the causative mutation, and comparative genomics are suggested to deepen in the analysis of QTL in turbot so they can be applied in marker assisted selection programs.

## Background

Growth related traits (e.g. body weight or length) constitute the main goal of genetic breeding programs in aquaculture. An increase in growth rate reduces production costs because it decreases the rearing time at farm facilities, thus increasing benefits for aquaculture companies [1]. The phenotype of these traits is generally associated to many genes of small effect according to the infinitesimal model [2], but also to few genes of high effect. There are two main approaches to address this fact: a) The identification of QTL or b) the identification of associated markers. QTL can be defined as DNA regions containing a gene or genes with relative high effect on a trait of interest [3]. The development of QTL studies through fine linkage maps may eventually lead to the identification of the particular gene/s underlying the trait, which promotes their use for breeding programs (GAS: Gene Assisted Selection). On the other hand, a trait associated marker is a neutral genome position in linkage disequilibrium with a gene/s underlying the trait. Thus, variation in the associated marker partially explains the phenotypic variation of the trait through a correlation with variation in the actual trait determining gene. Hence, associated markers can be also used in breeding programs (MAS: Marker Assisted Selection).

The effects of allele segregation at molecular markers throughout the genome can be used to determine the number, position and magnitude of QTL related to a particular trait [4]. For this purpose, the development of linkage genetic maps is essential [5]. Such maps have been developed for several commercial fish species [6].

To date, there are few studies on QTL affecting growth related traits in fish. Most of them were carried out in salmonids [7-9], and a number of significant QTL in the range from 2 to 4 was identified for each trait accounting for 20-25% of their phenotypic variation. Similar results were obtained in tilapia [10,11] and in sea bass [12-14]; in the latter case, QTL explained a higher proportion of the phenotypic variance (from 28% to 60%).

Turbot (*Scophthalmus maximus*) is a flatfish that has been intensively cultured during the last decade due to its great commercial value. With 22 haploid chromosomes (2 of them submetacentric, 11 subtelocentric and 9 telocentric) [15] and one of the smallest genomes among cultured fishes (< 800 Mb) [16], its production in Europe has increased from 3000 Tm in 1996 to 9246 Tm in 2009 [17]. As in many other cultured species, weight and length are traits of special interest. Size dispersion (both weight and length) in turbot culture, mainly due to sexual dimorphism since females largely outgrow males, is another key issue for its farming [18]. Only two studies about QTL detection have been carried out in this species. In the first one, a highly significant QTL for sex determination was identified [19], and in the second one a single QTL for body length was detected [20].

It has been suggested that flatfish (order Pleuronectiformes) growth is allometric [21]. These authors provided data in seven flatfish species from three different families: Soleidae, Bothidae and Citharidae, and clear departures from isometric growth were observed in four of them (two from Bothidae, one from Citharidae and one from Soleidae), whereas in three species (one from Bothidae and two from Soleidae) isometric growth appeared plausible. Therefore, growth model is expected to vary among different species, even considering species of the same order. As indicated, no data on species of Scophthalmidae, family to which turbot pertains, have been provided by these authors. A recent study in this species has shown that variation was not statistically different from isometric growth [22]. However, other studies in turbot have shown positive allometry associated with temperature [23], negative allometric growth being thus observed in late summer and autumn (temperature around 22°C), while positive allometric growth holds up in winter and spring (temperature around 7-10°C).

Martínez *et al*. developed a panel of homogeneously distributed markers ideal for QTL identification in turbot based on previous genetic mapping information [15,24,25]. The average distance between these markers in the consensus map is 14.43 cM, therefore meeting the minimum requirements for QTL analysis [26]. The recombination frequency ratio between males and females was determined to be 1.6:1 (female:male) in turbot [15], and consequently, the same linkage map can be used for both sexes without large errors.

Considering the commercial value of turbot and the availability of a suitable set of markers for QTL screening, it is possible to conduct a study on the detection of growth related QTL. The main objectives of this study are twofold: a) to detect and locate QTL affecting three growth-related traits in turbot: body weight, length and Fulton's condition factor, and b) to determine the association between microsatellite markers and growth related traits and compute their apparent effects.

## Methods

### Turbot Families

Eight families coming from two genetic breeding programs of turbot companies located in NW Spain were used in this study. Seven of them were obtained from Stolt Sea Farm SA (*FamPA-1, FamPA-3, FamPA-4, FamPA-6*, *FamAS-1*, *FamAS-2 *and *FamAS-3*) and one from Insuiña SA (*FamAP*).

Families were obtained following a three generation scheme starting from unrelated grandparents from natural populations of the Atlantic Ocean turbot fisheries area which led to full sibling pair analysis. Families were maintained under the same conditions at constant water temperature of 18°C in separated breeding tanks and using the standard culture procedure for this species [27]. Family size ranged from 85 to 113 individuals (Table 1).

**Table 1:** Descriptives for family mapping.

Family	N	NM	LG	ML	D	NMG
FamPA-1	93	100	26	1113.46	15.45	4.22
FamPA-3	85	193	26	1074.90	6.79	7.64
FamPA-4	91	90	23	1002.90	15.77	3.91
FamPA-6	90	98	26	1106.84	16.09	3.63
FamAS-1	100	104	22	1147.80	15.62	4.73
FamAS-2	100	98	22	1105.10	15.53	4.46
FamAS-3	100	99	22	1132.30	15.90	4.50
FamAP	113	101	23	1149.40	16.06	4.39

*N *is the number of evaluated individuals, *NM *is the number of microsatellites analysed, *LG *is the number of linkage groups, *ML *is the map length in cM, *D *is the average distance between markers in cM and *NMG *is the average number of markers per linkage group.

All experiments in this work have been reviewed and approved by the CETGA (Cluster de Acuicultura de Galicia) Committee on Bioethics.

### Trait measurements

Three growth related traits were evaluated: body weight (*We*), length (*Le*) and Fulton's condition factor (*FK*). Fulton's condition factor, developed by Fulton [28], is a measure of a fish fatness and can be computed as 100 * We/Le^3^, where *We *is the body weight of the fish in grams and *Le *is the length of the fish in centimeters. In our study, water temperature was maintained constant at 18°C. Then isometric growth in the studied families would be expected, and Fulton's factor should be a good approximation to fish condition, thus making it unnecessary to apply an allometric coefficient.

Pearson correlation coefficient (*r*) was used to determine the linear correlation between all pairs of analysed traits. Since differences in growth rate between sexes are significant only after 9 months post-hatching [18] and the age of evaluation was five months post-hatching for families *FamAS-1*, *FamAS-2*, *FamAS-3 *and *FamAP *and eight months post-hatching for families *FamPA-1*, *FamPA-3*, *FamPA-4 *and *FamPA-6*, the sex of evaluated individuals is not expected to influence the measured traits.

### Microsatellites and genetic map

Table 1 shows the number of microsatellites analysed for each family, the number of LGs they covered in the genetic map, the map length, the average distance between microsatellites and the average number of microsatellites per linkage group. Not all LGs were represented in all families because some markers were non-informative at specific families (e.g. both parents being homozygous) or there was only one marker available.

The panel of markers used for QTL identification was reported by Martinez *et al*. [19] and it is based on the consensus map by Bouza *et al*. [15], the mapping of ESTs by Bouza *et al*. [24] and the centromere mapping by Martinez *et al*. [25].

### Statistical procedures

#### QTL analyses

Two methodologies were used to detect QTL: Linear regression (LR) interval mapping using the *GridQTL *program [29,30] and maximum likelihood (ML) using the *QTLMap *program [31,32]. Each family was analysed separately, as differences in the age of evaluation and/or the maintenance of each family in separated tanks could distort the estimation of QTL effects if they were analysed together. The analysis of QTL in different families under different methods is expected to provide more confident estimations for the QTL existence (i.e. if it appears in different families or if it is detected with both methods). Also, a joint analysis of the 5- and the 8-month-old families, respectively, was done. All genotyping information (including grandparents) was used to obtain phase performance values.

*GridQTL *considers the linkage phase between markers in accordance with pedigree information. No fixed factor or covariate was included in the model except in *FamPA-3*, where sex information was available and it was considered to be a fixed factor. A single QTL was assumed at each LG and the default regression method (Haseman-Elston) was applied [33]. Chromosome-wide and genome wide significance thresholds were estimated by implementing a bootstrapping method at *p *= 0.05 and 0.01 [34], with a permutation test set to 10,000 iterations [35].

*QTLMap *detects QTL through interval mapping using ML estimates along the LG and the QTL position is estimated through the likelihood ratio [36,37]. For the computation of significant thresholds, 10,000 simulations were used under the null hypothesis (no QTL) for each trait and LG.

As previously suggested [7], chromosome-wide significance of QTL between 5% and 1% entailed its classification as suggestive, and significance below 1% entailed its classification as significant for both statistical methodologies applied. Genome-wide significances were also tested but no significant results were obtained.

### Association analysis

In order to investigate associations between phenotypic traits and molecular genotypes, a one-way ANOVA was performed for the progeny of each family, using the different genotypes of markers in the same LG where the QTL was detected. Each ANOVA provided also a corrected *R^2 ^*value that measured the reduction of the overall phenotypic variance of the trait due to the model fitting, thus providing the proportion of the variance of the trait that can be predictable from the given marker genotypes.

Before the ANOVA analysis, tests to check for normality (Shapiro-Wilk test) and homogeneity of variances (Levene test) were performed. Also a Bonferroni correction per LG was applied to the significance threshold for ANOVAs in order to cope with the problem of false positives in multiple comparisons.

## Results and discussion

This study represents the first wide QTL analysis for growth-related traits in turbot. A few studies have been addressed to detect growth-related QTL in fish and most of them have been carried out in salmonids [7-9], tilapia [10,11] and sea bass [12-14].

The power to detect QTL depends on the heritability of the trait, the recombination distance between the QTL and associated markers, the proportion of phenotypic variance explained by the QTL, the QTL allele frequencies and the sample size [38]. In this study, heritabilities assumed for weight, length and Fulton's factor were, respectively, 0.45, 0.3 and 0.2 [39], and the number of microsatellites and marker density is shown in Table 1. The number of microsatellites analysed in family *FamPA-3 *is higher than in other families because it was the reference family used for constructing the microsatellite genetic map of turbot [15]. The power of QTL analysis is limited, since only segregating QTL in one or both parents can be detected. In this study, the analysed families came from unrelated grandparents from different natural populations of the Atlantic area, where no significant genetic differences between populations were detected, excluding Baltic Sea populations [40,41]. In consequence, although the detected QTL might be representative of the genetic architecture of growth related traits in turbot, they should be verified in more turbot families previously to their use in breeding programs.

### Phenotypic values and correlations among growth-related traits

For each family, means (with standard error) for weight, length and Fulton's condition factor are shown in Table 2. The high dispersal of means across families is due to the family effect, to the different age of fishes and to some uncontrolled factors. This fact hinders the joint analysis of families.

**Table 2:** Descriptives for each family and analised trait.

Family	Weight (g)		Length (cm)		Fulton's factor	
	Mean	SEM	Mean	SEM	Mean	SEM
FamPA-1	39.79	1.19	12.64	0.13	1.93	0.02
FamPA-3	97.05	3.12	16.61	0.33	1.93	0.02
FamPA-4	119.28	3.89	17.66	0.20	2.09	0.02
FamPA-6	51.69	1.45	13.47	0.12	2.07	0.02
FamAS-1	46.31	0.90	12.81	0.08	2.18	0.02
FamAS-2	26.69	0.60	10.70	0.08	2.15	0.02
FamAS-3	31.62	0.72	11.33	0.09	2.14	0.02
FamAP	32.13	0.49	11.43	0.06	2.13	0.01

*SEM *is the standard error of the mean.

There was a highly significant positive correlation between weight and length with an average value of 0.890 (*p *< 0.001) (Additional file 1 Table S1). Also, a significant positive correlation, but lower, was observed between weight and condition factor (average value of 0.279 with *p *< 0.01). However, no significant correlation was detected between length and condition factor. Similar results were obtained in tilapia (*Oreochromis sp*.) with 0.91 for weight-length [10] and Atlantic salmon (*S. salar*) with 0.8 for body weight-condition factor [7]. Concerning condition factor and length, the non-significant correlation could suggest that loci influencing these traits may be segregating independently, although it should also be considered that linear correlations could be meaningless due to the cubic nature of the condition factor.

### Detection of QTL using GridQTL and QTLmap

Table 3 shows the QTL detected with each method for each trait. Although an important concordance was observed with both methods in the detected QTL, differences do exist, and the LR method (*GridQTL*) identified some QTL that were not detected using the ML one (*QTLMap*). Also ML provided, in general, higher significance for concordant QTL. Similar results were obtained by Kao [42], who detected that ML methods tend to be more powerful and differences between both methods tend to be larger as the marker interval becomes wider and the QTL position moves from boundary to the middle of an interval. He suggested the application of regression methods as the initial procedure to obtain preliminary results and then using the ML method in order to produce conclusive results. Also according to Imsland *et al*. [18], our results showed that sex does not have a significant effect after the analysis of *FamPA-3*.

**Table 3:** QTL detected with QTLMap (maximum likelihood) and GridQTL (regression interval mapping).

Method	Weight			Length			Fulton's factor		
	Family	LG	QTL position (cM)	Family	LG	QTL position (cM)	Family	LG	QTL position (cM)
**QTLMap**	FamPA-1	1	18.00	FamPA-1	15*	16.36	FamPA-1	2	45.00
		15*	18.36		16	38.37	FamPA-3	16*	0.00
		16	40.37	FamPA-3	1*	5.00	FamPA-4	3*	15.10
	FamPA-3	1	5.00		5*	20.31	FamPA-6	7	17.00
		5*	21.31		16	53.00	FamAS-1	4	60.00
		16*	52.00		17	13.00		7*	0.00
		17	15.00	FamPA-4	14*	30.92	FamAP	2	80.00
		20	24.00	FamPA-6	2	15.00			
	FamPA-4	14*	30.92	FamAS-2	6*	91.85			
		15	44.36		12*	49.57			
	FamPA-6	2	14.00	FamAP	12*	1.00			
	FamAS-2	6	91.85						
	FamAP	12	2.00						
									
**GridQTL**	FamPA-1	15	9.36	FamPA-1	15	12.36	FamPA-1	4*	0.00
		17	63.03		16	3.54		8	2.75
	FamPA-3	5*	26.18		17	63.03	FamPA-3	16*	0.00
		12*	52.90	FamPA-3	1	8.00		24	3.00
		16*	55.00		5	24.31	FamPA-4	3*	12.10
		17	20.00		12*	52.90	FamPA-6	11	23.05
		20	24.00	FamPA-4	14*	25.92	FamAS-1	4	70.00
	FamPA-4	14*	25.92	FamAS-1	13	30.00	FamAP	10	9.21
	FamPA-6	11	22.10	FamAS-2	1	0.00		16*	8.00
	FamAS-1	13	10.00		6	91.85		21	13.43
	FamAS-2	1	0.00		12	45.57			
		6	91.85	FamAS-3	3	36.10			
	FamAS-3	18	0.00	FamAP	12	1.00			
	FamAP	3	60.10						

A QTL can be significant (*) or suggestive.

QTL affecting all traits were detected in LG16 by both methods, and in some cases in more than one family, which evidences its high consistency. Likewise, both methods detected QTL for weight and length in LG5, LG6, LG14 and LG15 in only one family (excluding LG15 detected by ML for weight, in two families). In the case of LG1 in *FamPA-3 *and LG12 in *FamAP*, a QTL was detected for weight and length in one family by using the ML method (*QTLMap*), but only an effect on length was detected using the LR method (*GridQTL*).

Regarding Fulton's condition factor (*FK*), both methods detected a significant QTL in LG3. Suggestive QTL were detected in LG2 by ML in two families and in LG4 by LR in other two families. LR method detected a QTL in LG11 affecting weight and FK, but this QTL was not detected using the ML method.

The number of LGs with a significant QTL in the present study (from two to five depending on the trait and methodology used) is of the same order as reported in Arctic charr [8] or Asian seabass [12], using the experimental design of families of sibling pairs for QTL detection. Some QTL were found to affect two or more traits. This could be due to a gene/s being part of an early step in a metabolic route or acting in early stages of development affecting both traits, but also to linked genes affecting different traits. Similar positions of the QTL for weight-length and weight-condition factor were consistent with the positive correlations observed between these traits (0.890 and 0.279 respectively).

Joint analyses of the 5- or 8-month-old fishes, respectively, provided no significant or suggestive QTL, probably due to differences in strain genetic background that influences the degree of QTL expression as reported by Wringe *et al*. [43] or the existence of another uncontrolled factor in each family.

### Association analysis between microsatellite markers and growth-related traits

For each LG with a detected QTL (suggestive or significant), an association analysis was performed between the trait and all microsatellites in that LG (Tables 4, 5 and 6). The percentage of phenotypic variance explained by the marker (*R^2^*) was only calculated when the association was found significant. It could be expected that only the closest marker to the estimated position of QTL showed a significant association but in several cases, the associated marker was not always the closest one. This fact could be explained by several reasons: a) a low informative content of the real closest marker (this occurred for example in the case of ScmM1 at LG5, where the closest marker was Sma-USC56, but it had only two alleles and one of the parents was homozygous); b) a large extension area in linkage disequilibrium with the detected QTL could also cover several markers, resulting in positive associations between the trait and the genotypes at several markers (this occurred in the QTL detected for length in LG15 in *FamPA-1 *which, although showing a maximum *LR *value at 15.8 cM with *QTLMap*, covered a distance of many cM, including three markers) and c) the existence of a secondary segregating QTL, since markers could be in linkage disequilibrium with one or other QTL. Although no secondary QTL were detected in our study, this possibility should be taken into account for future studies. Also, it should be considered that the map positions in this study are not definitive, and the inclusion of new markers may produce some reorganizations.

**Table 4:** Significantly associated markers with weight.

Family	LG	Associated markers	Marker position	QTL Interval (QTLMap)	QTL Interval (GridQTL)	R^2^	NG
FamPA-1	15	Sma-USC214	8.36	8-36	0.4-11.4	8.00%	3
	15	Sma-USC32	23.32			13.60%	4
	15	Sma-USC211	24.22			6.80%	2
	16	Sma-USC256	35.36	35-46	-	7.00%	3
FamPA-3	1	Sma-USC15	0.00	3-7/9-11	-	13.70%	4
	5	ScmM1	24.71	10-30	16.3-30.3	11.20%	2
	16	Sma-USC223	53.01	45-56	49-55	12.90%	4
	16	Sma-USC50	55.84			12.90%	4
FamPA-4	14	Sma-USC220	8.60	20-38	5-28	17.90%	4
	14	Sma-USC82	25.40			10.60%	4
	14	Sma-USC63	30.50			10.60%	4
	15	Sma-USC149	45.20	40-46	-	12.90%	4
FamAS-2	6	SmaUSC-E7	92.40	90-93	-	8.00%	4

All positions and intervals are given in cM. *R^2 ^*is the corrected coefficient of determination from the ANOVA. *NG *is the number of genotypes observed for the marker in the full-sib family. Functionally annotated markers (BLAST; E-value < 10^-5^): Sma-USC223 (4SNc-Tudor Domain Protein; E-value: 7 × 10^-15^); Sma-USCE7 (similar to Fibroblast Growth Factor Receptor Substrate 2; E-value < 2 × 10^-14^).

**Table 5:** Significant associated markers detected on length.

Family	LG	Associated markers	Marker position	QTL Interval (QTLMap)	QTL Interval (GridQTL)	R^2^	NG
FamPA-1	15	Sma-USC214	8.36	8-36	8.3-30.4	8.50%	3
	15	Sma-USC32	23.32			12.90%	4
	15	Sma-USC211	24.22			7.70%	2
	16	Sma-USC256	35.36	35-49	3.54-7.07	8.20%	3
FamPA-3	5	ScmM1	24.71	10-30	17.3-28.3	10.30%	2
FamPA-4	14	Sma-USC220	8.60	20-38	8.9-31.9	16.20%	4
FamAS-2	6	SMA-USC-E7	92.40	90-93	90.9-93	10.90%	4
FamAP	12	3/9CA15	0.57	0-13	0-7	9.60%	4
	12	SmaUSC-E14	1.50			12.80%	4

All positions and intervals are given in cM. *R^2 ^*is the corrected coefficient of determination from the ANOVA. *NG *is the number of genotypes observed for the marker in the full-sib family. Functionally annotated markers (BLAST; E-value < 10^-5^): Sma-USCE7 (similar to Fibroblast Growth Factor Receptor Substrate 2; E-value < 2 × 10^-14^).

**Table 6:** Significant associated markers detected on Fulton's factor.

Family	LG	Associated markers	Marker position (cM)	QTL Interval (QTLMap)	QTL Interval (GridQTL)	R^2^	NG
FamPA-3	16	SmaUSC-E11	0.00	0-19	0-14	25.80%	4
FamPA-4	3	Sma-USC30	4.10	4-39	4-31	10.90%	2
	3	Sma-USC144	23.94			12.20%	4
FamPA-6	7	Sma-USC135	53.49	10-30	-	6.20%	2
	11	Sma-USC158	23.39	-	20-24	10.00%	4
FamAS-1	4	Sma-USC7	54.90	50-79	54-78	9.40%	2
	7	Sma4-14INRA	0.00	0-10	-	9.90%	4
FamAP	21	Sma-USC41	0.40	-	4-20	3.30%	2

All positions and intervals are given in cM. *R^2 ^*is the corrected coefficient of determination from the ANOVA. *NG *is the number of genotypes observed for the marker in the full-sib family. Functionally annotated markers (BLAST; E-value < 10^-5^): SmaUSC-E11 (Chloride Channel 3; 2 × 10^-19^); Sma-USC7 (Insulin-like Growth Factor 1 Receptor; E-value: 0).

Regarding weight (Table 4), the highest *R^2 ^*(17.90%) was associated to Sma-USC220 in LG14, where a significant QTL was detected by both *QTLMap *and *GridQTL *in family *FamPA-1*. Sma-USC15 and Sma-USC32 provided also a high *R^2 ^*(13.70% and 13.60%), being located respectively in LG1 in *FamPA-3 *(where a QTL was detected only with *QTLMap*) and in LG15 in *FamPA-1 *(where a QTL was detected by both methods).

Considering length (Table 5), the highest *R^2 ^*(16.20%) was explained again by Sma-USC220, with a significant QTL detected by both methods. Marker SmaUSC32 provided also a high *R^2 ^*(12.90%) with a QTL again detected with both methods.

For Fulton's factor (Table 6), the highest *R^2 ^*(25.8%) was associated to marker SmaUSC-E11, when analysing LG16 in family *FamPA-3*. It was associated to a significant QTL both detected by *QTLMap *and *GridQTL*. Marker Sma-USC144 in LG3 in family *FamPA-4 *also showed a high *R^2 ^*(12.20%) in association to a significant QTL detected by *QTLMap*.

In general, the proportion of phenotypic variance explained by tightest linked markers to the significant QTL for weight varied from 8 to 13.6%, from 8.2 to 12.9% for length and from 3.3 to 25.8% for Fulton's condition factor. Similar results were obtained in salmon by Reid *et al*. [7]. In our study, it was remarkable the case of Sma-USC32 marker, associated to a QTL in LG15, which showed the maximum *R^2 ^*for both weight and length, thus indicating a high association with the trait and the possibility of using it for MAS. Also, as both *QTLmap *and *GridQTL *agreed on the detection and positioning of 11 QTL, these are expected not to be false positives. Moreover, association analyses with microsatellite markers in these LGs were significant, giving additional support to our findings. No association was found in several LGs with detected QTL, probably due to the low informativeness of involved markers. These were some cases for weight in LGs 2, 3, 11, 12, 13, 17, 18 and 20; for length in LGs 1, 2, 3, 13 and 17; and for Fulton's factor in LGs 8, 10 and 24.

Most significant associated markers with the QTL detected on growth traits were anonymous microsatellites isolated from partial genomic libraries [15], since only a small number of gene-linked markers have been mapped in the turbot to date [24]. Interestingly, four of them were functionally annotated as growth-related genes, therefore representing putative candidate genes for the traits under study to be further evaluated in other turbot families. Thus, the anonymous locus SmaUSC223 (LG16: 53.01 cM; Table 4), which explained almost 13% of phenotypic variance for a weight QTL, could be annotated as 4SNc-Tudor domain protein. This gene belongs to an evolutionarily conserved family of key regulators of gene expression in eukaryotes, with relevant role in both transcription and pre-mRNA splicing, but also in RNA-induced gene silencing and cell proliferation *via *activating transcription factors [44]. Also, we detected other three markers associated to functionally annotated growth-related genes: i) SmaUSC-E11 (LG16: 0.00 cM; Table 6), which explained close to 26% of phenotypic variance for a Fulton's factor QTL, was annotated as Chloride Channel-3. This gene may be indirectly related to growth, as pointed out in several studies that revealed an influence of water salinity on fish development and growth, from early embryogenesis to adult stages [45,46]; ii) SmaUSC7 (LG4: 54.9 cM; Table 6), which explained close to 10% of phenotypic variance for a Fulton's factor QTL, was annotated as Insulin-like Growth Factor 1 Receptor, a highly relevant growth-related gene across animal species, including fish [47,48]; and iii) SmaUSC-E7 (LG6: 92.4 cM; Tables 4 and 5), significantly associated with weight and length QTL, was annotated as Fibroblast Growth Factor Receptor Substrate 2, a gene tightly associated with muscle mass growth in teleosts [48]. All these turbot candidate sequence tags should be checked across new families to confirm the detected association and analysed for gene expression using Q-PCR in individuals or families showing marked differences in growth rate. Searching for new markers around the detected QTL, especially for those gene-linked ones, will be essential to refine marker-associations within each family and to provide new putative candidates for a functional explanation.

Another way to check QTL associations and look for candidate genes is comparative mapping [49,50]. In a study by Bouza *et al*. [15], syntenic relationships were observed between sequences of turbot and the model fish *Tetraodon nigroviridis*, suggesting true homology of those associated regions. Thus, a collinear syntenic block was identified between turbot LG16 and the chromosome 19 of *T. nigroviridis *(*Tni*19) based on three loci (Sma-USC136, Sma-USC285 and Sma-USC223; ~10 cM interval). In the present work, a QTL for weight was detected in LG16 associated to the closely linked markers (~2 cM) Sma-USC223 and Sma-USC50 (Table 4), although this latter one did not show sequence similarity against *T. nigroviridis *genome. The study of this syntenic block in *T. nigroviridis *could provide information about candidate genes for the detected QTL, given the high synteny conservation among phylogenetically diverse fish genomes [50]. Therefore, we decided to examine the microsyntenic region from 1,000 kbp to 5,000 kbp in *Tni*19 in more detail, trying to cover the small genetic distance between the two weight-QTL associated markers, SmaUSC223 and Sma-USC50, assuming a physical-genetic ratio of about 0,5 kbp/cM [15]. A gene list from this region of *Tni*19 was obtained using the BioMart tool from Ensembl. Several of these genes appeared associated to GO terms related to growth regulation and cell proliferation (GO:0030308; GO:0001558; GO:0040008; GO:0042127), some of them very close to the syntenic position of the QTL associated marker SmaUSC223 (e.g. CD9 molecule; CCND2-Cyclin D2; HCLS1-B-cell translocation gene 1, anti-proliferative; BTG1-Hematopoietic cell-specific Lyn substrate 1; FOXM1-forkhead box M1; PTN-Pleiotrophin; PAWR-Apoptosis WT1 regulator; LAMB1-Laminin, beta 1; NRCAM-Neuronal cell adhesion molecule; ADIPOR2-Adiponectin receptor 2; SEMA3A-Semaphorin 3A; CHPT1-Choline phosphotransferase 1). The list also included some relevant growth-related candidate genes previously reported in fish (e.g. HGF-Hepatocyte Growth Factor; FGF6-Fibroblast Growth Factor 6; IGF1-Insuline Growth Factor 1) [48,50]. Putting forward new genetic markers for these candidate genes and testing them in turbot families will be essential to go further in the functional explanation of the detected associations, as in the previous marker-associated approach.

## Conclusions

The use of different statistical methods (LR interval mapping and ML) for QTL detection provided consistent results, the use of several families to look for the repeatability of detected QTL also being of great advantage. Although the age of evaluation and the culture conditions have been controlled, a high variation for mean traits was observed even between families of similar age, thus making it difficult to evaluate QTL effects and the estimation of heritability (data not shown). This high variation can be due to uncontrolled factors. Finer mapping of detected QTL could lead to the identification of candidate genes useful for GAS. Favoured by an increase of marker density, another approach could be looking for tightly linked markers that can be used in MAS. In addition, the use of genetically divergent grandparents in new studies would provide higher accuracy for estimation of QTL effects, also allowing the estimation of QTL gene frequencies. Finally, comparative genomics in homologous regions with model fish emerges as a useful strategy for verification of the identified QTL and to look for candidate genes.

## Authors' contributions

ES was responsible for the final version of QTL and marker analysis and the writing of the article. AC was responsible for a first version of the QTL analysis. MAT and JF were responsible for overseeing QTL and marker analysis, as well as paper supervision. SC was responsible for fish rearing and phenotypic trait measuring. BP, CB and MH performed genotyping on all families and comparative mapping analysis. PM was responsible for the whole work and managed all tasks. All authors have read and approved the final manuscript.

## Supplementary Material

Additional file 1 **Pairwise correlation between traits**. Click here for file
