# Supplementary material for: Detection of growth-related QTL in turbot (Scophthalmus maximus)
Source: BMC Genomics. 2011 Sep 29;12:473. doi: 10.1186/1471-2164-12-473 (PMC3195100; doi:10.1186/1471-2164-12-473)
Supplement: Additional file 1 — Pairwise correlation between traits. [file 1471-2164-12-473-S1.PDF]

Pairwise correlations between traits.

Family	Correlations		
	**We-Le**	**We-FK**	**Le-FK**
Fam01	0.956***	-0.005	-0.279
Fam03	0.542***	0.266*	-0.079
Fam04	0.963***	0.433***	0.259*
Fam06	0.950***	0.341***	0.057
FamAS-1	0.929***	0.267***	-0.094
FamAS-2	0.942***	0.350***	0.042
FamAS-3	0.928***	0.234***	-0.128
FamAP	0.906***	0.347***	-0.072
**Average**	0.890***	0.279**	-0.037

Weight (We), length (Le) and Fulton´s factor (FK). P-value: *(<0.05) **(<0.001) ***(<0.001).
